# Mortality Risk and Decompensation in Hospitalized Patients with Non-Alcoholic Liver Cirrhosis: Implications for Disease Management

**DOI:** 10.3390/ijerph18020606

**Published:** 2021-01-12

**Authors:** Ming-Shun Hsieh, Kung-Chuan Cheng, Meng-Lun Hsieh, Jen-Huai Chiang, Vivian Chia-Rong Hsieh

**Affiliations:** 1 Department of Emergency Medicine, Taipei Veterans General Hospital, Taoyuan Branch, Taoyuan 330, Taiwan; edmingshun@gmail.com; 2 Department of Emergency Medicine, Taipei Veterans General Hospital, Taipei 11217, Taiwan; 3 School of Medicine, National Yang-Ming University, Taipei 11221, Taiwan; 4 Division of Colorectal Surgery, Department of Surgery, Kaohsiung Chang Gung Memorial Hospital, Kaohsiung 83301, Taiwan; topguncheng@cgmh.org.tw; 5 Department of Education, Taichung Veterans General Hospital, Taichung 40705, Taiwan; morganpolo@gmail.com; 6 Department of Health Services Administration, College of Public Health, China Medical University, Taichung 406040, Taiwan; zinvii@gmail.com

**Keywords:** liver cirrhosis, nonalcoholic, chronic hepatitis B, chronic hepatitis C, complications, varices, ascites, hepatic encephalopathy, cause of death, mortality, disease management

## Abstract

Here we aimed to assess the mortality risk and distribution of deaths from different complications and etiologies for non-alcoholic liver cirrhosis (NALC) adult inpatients and compare them with that of the general hospitalized adult population. Hospitalized patients with a primary diagnosis of NALC and aged between 30 and 80 years of age from 1999 to 2010 were identified using a population-based administrative claims database in Taiwan. They were matched with a general, non-NALC population of hospitalized patients. Causes of death considered were variceal hemorrhage, ascites, hepatic encephalopathy, spontaneous bacterial peritonitis, hepatocellular carcinoma, jaundice, and hepatorenal syndrome. A total of 109,128 NALC inpatients were included and then matched with 109,128 inpatients without NALC. Overall mortality rates were 21.2 (95% CI: 21.0–21.4) and 6.27 (95% CI: 6.17–6.37) per 100 person-years, respectively. Among complications that caused death in NALC patients, variceal hemorrhage was the most common (23.7%, 11.9 per 100 person-years), followed by ascites (20.9%, 10.4 per 100 person-years) and encephalopathy (18.4%, 9.21 per 100 person-years). Among all etiologies, mortality rates were highest for NALC patients with HBV infection (43.7%, 21.8 per 100 person-years), followed by HBV-HCV coinfection (41.8%, 20.9 per 100 person-years), HCV infection (41.2%, 20.6 per 100 person-years), and NAFLD (35.9%, 17.9 per 100 person-years). In this study, we demonstrated that mortality risks in NALC patients may differ with their etiology and their subsequent complications. Patients’ care plans, thus, should be formulated accordingly.

## 1. Introduction

Cirrhosis of the liver is a highly prevalent disease in Asian-Pacific countries, accounting for an estimated of 328,000 deaths per year [1]. The main causes of this condition in western countries are primarily alcohol intake and non-alcoholic fatty liver disease (NAFLD), whereas viral hepatitis B (HBV) infection is the most common cause in most parts of Asia such as Taiwan [2]. Taiwan still has a relatively high proportion of HBV- and HCV-infected population despite its decline since the implementation of the national viral hepatitis therapy programs [2,3]. Additionally, more health-damaging lifestyles such as high sugar intake and physical inactivity are observed, leading to metabolic diseases and fatty liver which are significant factors in the increasing prevalence of non-alcoholic liver cirrhosis (NALC) in this east Asian country [4]. 

The 5-year survival in cirrhotic patients can be as low as 31% following admission, and the etiologies for cirrhosis-related mortality can greatly vary with context [1,5,6]. Nevertheless, the majority of deaths among cirrhosis patients are due to complications developed in transition to the decompensation state. Patients with decompensated cirrhosis have a very high risk of mortality, particularly from conditions like variceal hemorrhage, ascites, and encephalopathy. These complications are most likely consequences of portal hypertension characterized by escalated blood pressure within the portal venous system. Existing literature showed that among chronic hepatitis B-related cirrhosis patients, approximately 8.5% could develop variceal bleeding, 3.1% ascites, 19.3% spontaneous bacterial peritonitis (SBP) and 29.0% encephalopathy [7,8,9]. Although there are estimates of mortality risks available for these patients, no clear separation of cirrhotic-mortality according to cause (by complication) and etiology is currently available. More importantly, there is no recent data to provide evidence into the likelihoods of mortality from various complications in a real-world setting in order to guide clinicians regarding patients’ disease management.

Many studies have already assessed the risk of mortality and hepatocellular carcinoma (HCC) in cirrhosis patients [7,10]. However, to date, the excess mortality rates from different complications and etiologies of these patients have not yet been evaluated. Thus, the purpose of this study is to report population-based estimates on the excess mortality rates for NALC patients of different etiologies and complications, and compare their distributions and risks of mortality with subjects without NALC. Factors that influence their mortality risk will also be assessed.

## 2. Materials and Methods

### 2.1. Data Source and Study Design

**Figure 1:**
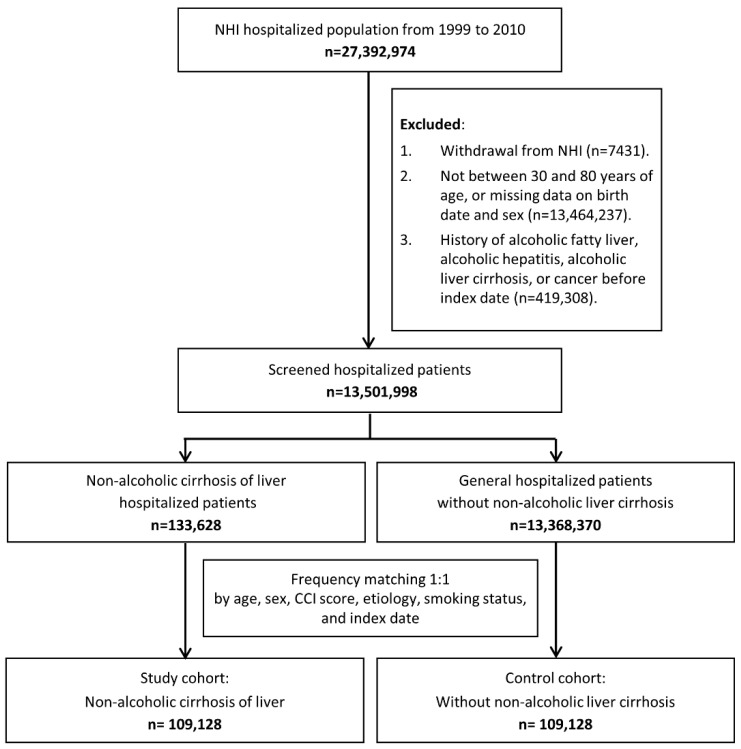
Subject selection process flowchart.

We used a population-based administrative claims database in Taiwan, the National Health Insurance Research Database (NHIRD), to identify all inpatients aged between 30 and 80 years of age with a primary diagnosis of NALC (International Classification of Diseases, Ninth Revision, Clinical Modification (ICD-9-CM) codes 571.5 and 571.6) within a period of 12 years from 1 January 1999 to 31 December 2010. NHIRD is an administrative claims-based database for the country’s single-payer national health insurance. It holds a comprehensive set of claims data for >99% of the population and their uptake of services including outpatient, hospitalization, and emergency care, as well as any associated prescriptions and laboratory tests. Patients with the following characteristics were excluded from the study: (a) pre-diagnosed with cancer before index date, or (b) history of alcoholic-related conditions like alcoholic fatty liver (571.0), alcoholic hepatitis (571.1), alcoholic liver cirrhosis (571.2), and alcoholic liver damage (571.3). Our process for subject selection has been illustrated in Figure 1.

We designed a retrospective cohort study in which the NALC subjects were matched 1:1 to the general inpatient population without any diagnosis of NALC (control cohort). The two cohorts were matched based on age, sex, Charlson Comorbidity Index (CCI), etiology, smoking status, and index date (i.e., incident date of hospital admission). Etiologies considered in this context were chronic viral hepatitis B (HBV) and C (HCV), HBV-HCV coinfection (HBV+HCV), and non-alcoholic fatty liver disease (NAFLD). NAFLD was defined if subject had a diagnostic code of ICD-9-CM code 571.8 on their medical record before index date. Since lifestyle factors such as smoking cannot be determined in a claims database but can be a confounding factor for mortality, we used the status of chronic obstructive pulmonary disease (COPD) (ICD-9-CM: 490, 491, 492, 494, 496) as a proxy for long term smoking. All study subjects were observed until 31 December 2011, event of death, or withdrawal from the National Health Insurance, whichever occurred first.

### 2.2. Primary Outcome

Mortality was our outcome of interest; it was defined up to two weeks following patient’s discharge from hospital. Various causes of death common to patients with liver cirrhosis were classified according to their last admission records (before death) and listed diagnostic code(s): all-cause, variceal hemorrhage/bleeding (ICD-9-CM: 456.0, 456.2, 578.0, 578.1, 578.9), ascites (568.82, 789.5), hepatic encephalopathy (HE) (572.2), SBP (567.2, 567.8, 567.9), HCC (155.0, 155.2), jaundice (782.4), and hepatorenal syndrome (HRS) (572.4). Jaundice was treated here as an acute decompensation and not secondary to an acute-on-chronic liver failure.

As admitted patients have higher probability of multiple and more severe conditions, comorbidities were also considered and defined in the form of CCI score and chronic smoking (i.e., COPD). All considered etiologies and comorbidities were defined in the subjects’ medical history one year prior to their index dates.

### 2.3. Statistical Aanalysis

For analysis, continuous data are expressed using means and standard deviations, whereas categorical data are presented as counts and percentages. Crude mortality rates are expressed in rates (per 100 person-years, PYs) with 95% confidence intervals (CIs). Chi-squared test was used to test the difference in the proportion of deaths between two cohorts. Kaplan-Meier curve was used to show the cumulative survival probability of the study cohorts over time while log-rank test was performed to test the significance of difference between the two survival curves. Finally, Cox proportional hazards model was used to estimate hazard ratios (HRs) for mortality after adjusting for confounding factors. A two-sided *p*-value of <0.05 was considered statistically significant.

### 2.4. Subgroup Analysis

To test if risks of death differed with etiology and complication, subgroup analysis was performed for both the excess mortality risks and adjusted hazard ratios for mortality. HBV and HCV were considered as etiologies since they are the most prevalent hepatitis infections in Taiwan. NAFLD was chosen because it has become increasingly prevalent as a result of changing lifestyles and diet leading up to metabolic syndromes such as obesity and diabetes mellitus.

### 2.5. Ethical Considerations

This study was approved by the research ethics committee at China Medical University and Hospital (CMUH107-REC2-105). Informed consent was waived due to the retrospective nature of this study and our use of secondary data for analyses.

## 3. Results

### 3.1. Baseline Characteristics of the Study Cohorts

Our final cohorts comprised 109,128 NALC subjects and 109,128 matched control patients without NALC. Their total follow-up was 355,486 PYs and 583,423 PYs, respectively. Baseline attributes of the two cohorts were similar post-matching: male (68.3%), mean age of 59.4 years, etiology (HBV-only (13.6%), HCV-only (10.9%), HBV-HCV coinfection (1.31%), and NAFLD (3.80%)) and CCI scores (1: 56.1%; ≥2: 43.9%). However, as expected, the prevalence of decompensation was much higher in NALC patients. General characteristics of the study cohorts are presented in Table 1.

### 3.2. Excess Mortality Risk from Decompensation

**Figure 2:**
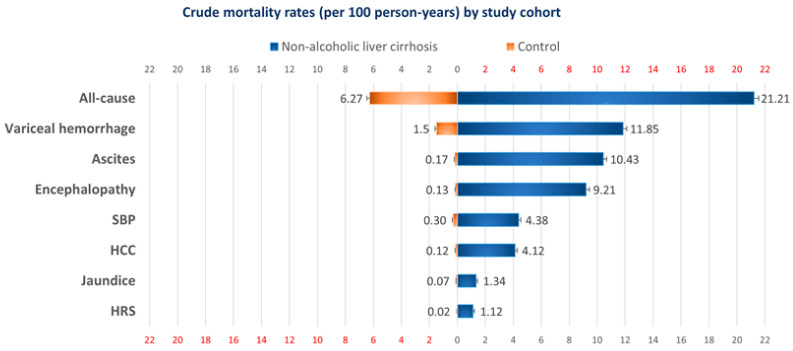
An illustration depicting the difference in excess mortality risks between non-alcoholic liver cirrhosis and control patients.

Table 2 illustrates the proportions and crude mortality rates for the cohorts with and without NALC. In general, 42.4% (46,297 of 109,128) of inpatients with cirrhosis died compared with 12.5% (13,684 of 109,128) in their counterparts during the follow-up period. Overall mortality rates were 21.2 (95% CI: 21.0–21.4) and 6.27 (95% CI: 6.17–6.37) per 100 PYs, respectively, and the difference was statistically significant (*p* < 0.0001). Among complications that caused death in NALC patients, variceal hemorrhage was the most common (23.7%, 11.9 per 100 PYs), followed by ascites (20.9%, 10.4 per 100 PYs) and encephalopathy (18.4%, 9.21 per 100 PYs). All other considered causes of death accounted for mortality of less than 10% each in NALC patients: SBP 8.8%, HCC 8.2%, jaundice 2.7%, and HRS 2.2%. Figure 2 provides a graphic representation of the comparison of mortality rates between the two cohorts.

**Figure 3:**
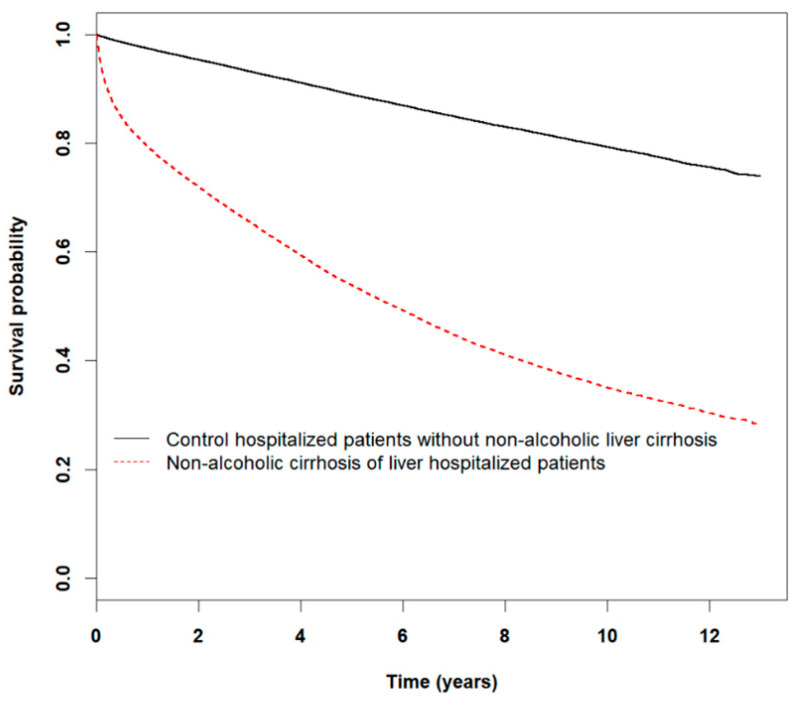
Kaplan-Meier curves for all-cause mortality for study and control cohorts.

For the control cohort, the number of deaths from decompensation was negligible, with only variceal hemorrhage with a slightly higher mortality rate of 1.50 per 100 PYs (3.0%). Kaplan-Meier curves for all-cause mortality also highlight the significantly lower cumulative survival probability in the NALC cohort relative to the control (log-rank test, *p* < 0.0001). The sharp decrease within the first year of follow-up was especially noticeable (Figure 3).

Table 3 presents the excess risk of death further stratified by etiology among NALC patients. Overall mortality rates were highest for NALC patients with HBV-only etiology (43.7%, 21.8 per 100 PYs), followed by HBV-HCV coinfection (41.8%, 20.9 per 100 PYs), HCV-only (41.2%, 20.6 per 100 PYs), and lastly, NAFLD (35.9%, 17.9 per 100 PYs). Among the complications, variceal hemorrhage, ascites, and encephalopathy were the top three causes of death across all etiologies. We also noticed that although mortality rates were generally lowest in NAFLD among the four etiologies, they were still considerably higher than that of the control inpatient cohort.

### 3.3. Adjusted Mortality Risk in Patient Subgroups

To examine under which attributes do NALC patients have significantly elevated mortality risk than non-NALC patients, we stratified our subjects into subgroups of etiology, sex, and age (Table 4).

We first compared their all-cause mortality risk after adjusting for etiology, age, sex, CCI score, smoking status, and index date. We found that NALC patients of HBV-only etiology had the highest adjusted hazard ratios (aHRs) versus the control patients within the etiology subgroup (aHR: 8.38 (95% CI: 7.87–8.93)), and <40 years of age had the highest aHR among all age groups (aHR: 13.3 (95% CI: 11.9–15.0)). This suggests that among hospitalized patients with HBV infection, those with NALC are 8.38 times more likely to die from any cause compared with those without NALC; among patients <40 years of age, those with NALC are 13.3 times more likely to die from any cause compared with those without NALC. It is also important to note that, among inpatients with HBV-HCV coinfection, the relative risk was also significantly high (aHR: 6.69; 95% CI: 5.53–8.10).

We subsequently presented the adjusted risks of cause-specific mortality (variceal hemorrhage, ascites, encephalopathy, SBP, HCC, jaundice, HRS). Results indicate that the risk of death from variceal hemorrhage in NALC patients with HBV was 16.1 times higher than non-NALC patients with HBV (95% CI: 14.3–18.0). Similarly, aHR for death by ascites was 83.3 times higher in NALC patients with NAFLD compared with non-NALC patients with NAFLD (95% CI: 45.9–151.0). When examining encephalopathy as the cause of death, females with NALC had an aHR of 164.5 (95% CI: 127.3–212.6) relative to females without NALC. The increased complication-specific mortality risks for NALC patients compared with non-NALC individuals were also more profound in the younger age groups of <40, 40–49, and 50–59. This confirmed that NALC ubiquitously elevated patient’s mortality risk despite the cause and that the mortality risk can vary with patients’ etiology, age and sex.

## 4. Discussion

### 4.1. Main Findings

In this population-based retrospective cohort study, we demonstrated that variceal bleeding, ascites, and encephalopathy can potentially be the three leading causes of mortality among NALC patients. Our study also confirmed that the mortality risk varies with patient’s etiology, age, and sex, as well as the complication developed in these patients when advancing to the decompensation state. Specifically, patients of HBV etiology had the highest excess risk of mortality from decompensation amongst all etiology subgroups (HBV, HCV, HBV + HCV, and NAFLD). This may be partly due to the insidious nature of clinical course and disease progression in HBV patients which is different from other etiologies, subsequently elevating their risk of death. Also, it is believed that the prognosis for most chronically HCV-infected patients tend to be relatively nonthreatening over a period of time despite their clinical progression [11]. Elevated complication-specific mortality risks for NALC patients relative to non-NALC individuals were also more profound in <40 years, 40–49 years and 50–59 years age groups.

### 4.2. Comparison with Other Studies

A similar investigation was conducted in England looking into deaths by etiology in liver cirrhosis patients relative to the general population [12]. In a total of 5118 cirrhosis patients, crude (all-cause) mortality rate for viral hepatitis-related cirrhosis was 14.1 per 100 PYs which is less than what was observed in our population (21.2 per 100 PYs). This may be attributable to the hospitalized status of our study cohorts. The increased mortality risk in cirrhosis patients relative to non-cirrhosis patients was also consistent with our findings but was more considerable in the English study (6-fold vs. 3-fold). Nevertheless, it is important to note that results from our study should be interpreted differently since we considered the complications as our possible causes of death while removing alcoholic-related factors.

Tapper and Parikh (2018) found in their US cohort study that age-adjusted cirrhosis mortality was 12.18/100,000 population; this rate has been escalating and the greatest rate of change was attributed to alcohol use in young people [13]. Although their outcome of interest is similar to that of this study, they examined distribution of mortality risk by race and geographical region which cannot be directly compared with our results. Also, unlike this study, they included alcohol-related cirrhosis. In another US study examining the trends in mortality of cirrhosis patients, mortality rate in viral hepatitis-related cirrhosis was 9.0 per 100 PYs which is much lower than what we estimated due to the reason noted above. However, similar to our observation, they detected a higher mortality risk from ascites (12.4/100 PYs), HE (5.3/100 PYs), and variceal bleeding (2.9/100 PYs) among the cirrhosis-related complications [14].

For NALC patients, we detected a very high probability of death within the first year following their hospital admission. This agrees with previous evidence from England which suggested that mortality could be as high as 8 times more than that in the general population at one year [15]. Another Danish study also showed that up to 38% of cirrhosis patients die within the first year of follow-up [16].

We also found that NAFLD and HBV-HCV co-infected patients had very high risks of death from complications even after accounting for confounding factors. While there is lack of evidence on the odds of death in NAFLD cirrhosis patients, much evidence did indicate an increased risk of disease progression into cirrhosis, decompensation, and HCC in HBV-HCV co-infected cases [17,18,19]. We included NAFLD in this study and demonstrated its plausible impact on the mortality risk in NALC patients. This is crucial for Asian countries since it has become an emerging epidemic imposed by dietary and lifestyle changes with serious health outcomes [20].

Among complications that led to mortality, variceal hemorrhage was most commonly observed, followed by ascites and encephalopathy. A cohort study conducted in Denmark confirmed that 56% of liver cirrhosis hospitalized patients die in the first year from associated conditions such as esophageal varices and hepatic coma [16]. Similar to our study, they also noticed increased mortality in patients of chronic hepatitis etiology. A descriptive study in India demonstrated that the most common cause of death among cirrhosis patients was HE (50%) followed by gastrointestinal bleeding (27%), and that HBV is a prevalent etiological factor in this Asian population [21]. Moreover, results from a Korean study have confirmed that mortality rate of decompensated cirrhosis patients is even significantly greater than patients with cancers [22].

Many of the complications of cirrhosis are the consequence of portal hypertension. Formation of varices may be ensued, as well as vascular and other abnormalities that contribute to the pathogenesis of ascites and other complications like encephalopathy [23]. All these are prognostic factors that can predispose to premature mortality in patients with cirrhosis.

For instance, variceal bleeding is a frequent condition in decompensated cirrhosis with a higher risk of mortality than the other causes of gastrointestinal bleeding [24]. Indeed, we showed that variceal bleeding is the primary cause of death among all cirrhosis-related complications, which agrees with the clinical staging of cirrhosis and its corresponding prognosis [25]. High mortality attributed to this complication is mainly due to the high fatality of its initial episodes, as well as the high probability for recurrent bleeding [24,25,26]. High mortality in subjects with ascites and hepatic encephalopathy is also supported by other studies [12,27,28].

### 4.3. Strengths and Limitations

For our study, we specifically targeted NALC patients, unlike many previous studies which dealt primarily with alcoholic cirrhosis. To the best of our knowledge, this is the first population-based cohort study that has been conducted to investigate cause-specific deaths by etiology in NALC patients. Furthermore, past studies were either conducted in hospital-based settings which could lead to overestimation of mortality, or they did not explore the complications as specific causes of deaths and their risks; they mostly examined neoplasm-, metabolic-, or circulatory-related factors as causes of death [12,15,16,21,29]. Moreover, NAFLD was considered as an etiology in our study which was rarely observed in earlier studies. This provides novelty and an update to our current knowledge about NALC epidemiology in adherence with modern lifestyle and social transition. We also considered the impact of ill-status baseline of hospitalized patients on their prognosis; this is why we performed rigorous matching. One of the criteria was patients’ CCI that took into account the effect of other comorbidities such as cardiovascular diseases and metastatic solid tumor.

Nevertheless, this study is not without limitations. Clear separation of liver disease by etiology is unfeasible even in developed economies as suggested by a previous study [1]. For our analysis, the occurrence of decompensation is not mutually exclusive from one another for each patient, i.e., we identified each condition when its corresponding code is found in its admission record, irrespective of its order (primary, secondary, or tertiary diagnosis). Hence, one may be counted more than once when we stratified the analysis into subgroups by type of decompensation. Also, for the purpose of our study, we did not examine closely the heterogeneity within the HBV-HCV co-infected population which may be affecting the viral genotypes and/or activity status of the viruses. Lastly, there are several caveats using claims-based administrative data: First, laboratory details such as bilirubin levels, prothrombin time or viral load were not available in the administrative data sources to verify patients’ diagnosis and classify the severity of disease (i.e., Child-Pugh-Turcotte score); secondly, NAFLD stage, which could influence cirrhosis prognosis, could not be defined without the presence of biopsy or histological data; and thirdly, our estimates of cirrhotic mortality should be interpreted with caution since they are based on hospitalized cases (i.e., overestimation). Many cases of liver cirrhosis in the early stages are asymptomatic and often diagnosed. COPD as a surrogate for smoking status could potentially misclassify or underestimate the prevalence of smoking in the subject sample since only those that were chronic smokers and developed COPD would be identified.

### 4.4. Implications on Disease Management

Our results have important implications to disease management for NALC patients. Our evidence on the varying mortality risk among hospitalized NALC patients should caution clinicians to be vigilant while caring inpatients of various etiologies (e.g., HBV, NAFLD) and age groups. Moreover, different clinical measures should be taken to prevent different complications, most likely in the order of their severity and risk of death. We hope that the evidence presented in this study can help further improve quality of care by guiding clinicians in their practice under the real-world setting.

## 5. Conclusions

In conclusion, results from this population-based retrospective cohort study demonstrated the significant elevated risk of mortality among NALC patients compared with general inpatients. Specifically, variceal bleeding, ascites, and encephalopathy can potentially be the three leading causes of mortality among these patients. To ensure quality of care and effective management of disease, patient etiologies and potential complications must also be taken into consideration when formulating patients’ care plans.

## Figures and Tables

**Table 1:** Baseline characteristics of the study and control cohorts.

Variable	Non-Alcoholic Liver Cirrhosis		Control	
	*n* = 109,128		*n* = 109,128	
	*n*	%	*n*	%
Sex				
Female	34,636	31.7	34,636	31.7
Male	74,492	68.3	74,492	68.3
Age (years)				
<40	8520	7.80	8520	7.80
40–49	20,078	18.4	20,078	18.4
50–59	24,915	22.8	24,915	22.8
60–69	27,444	25.2	27,444	25.2
≥70	28,171	25.8	28,171	25.8
Mean ± SD	59.4 ± 12.6		59.4 ± 12.7	
Etiology				
HBV only	14,838	13.6	14,838	13.6
HCV only	11,861	10.9	11,861	10.9
HBV+HCV	1425	1.31	1425	1.31
NAFLD	4148	3.80	4148	3.80
Comorbidities				
Smoking status (COPD)	9677	8.87	9677	8.87
CCI score				
1	61,208	56.1	61,208	56.1
≥2	47,920	43.9	47,920	43.9
Decompensation				
Variceal hemorrhage	50,451	46.2	12,234	11.2
Ascites	41,792	38.3	1359	1.3
Encephalopathy	34,460	31.6	989	0.9
SBP	16,176	14.8	2673	2.5
HCC	15,777	14.5	615	0.6
Jaundice	5876	5.4	1135	1.0
HRS	3478	3.2	154	0.1

Abbreviations: SD—standard deviation; HBV—hepatitis B virus; HCV—hepatitis C virus; NAFLD—non-alcoholic fatty liver disease; COPD—chronic obstructive pulmonary disease; CCI—Charlson comorbidity index; SBP—spontaneous bacterial peritonitis; HCC—hepatocellular carcinoma; HRS—hepatorenal syndrome.

**Table 2:** Crude mortality rates per 100 person-years for the cirrhosis and control cohorts.

Cause of Death	Non-Alcoholic Liver Cirrhosis *n* = 109,128		Control *n* = 109,128		*p* Value
	(355,486 Person-Years)		(583,423 Person-Years)		
	Deaths (*n*,%)	Rate (95% CI)	Deaths (*n*,%)	Rate (95% CI)	
All-cause	46,297 (42.4)	21.2 (21.0–21.4)	13,684 (12.5)	6.27 (6.17–6.37)	<0.0001
Variceal hemorrhage	25,874 (23.7)	11.9 (11.7–12.0)	3278 (3.0)	1.50 (1.45–1.55)	<0.0001
Ascites	22,778 (20.9)	10.4 (10.3–10.6)	373 (0.3)	0.17 (0.15–0.19)	<0.0001
Encephalopathy	20,107 (18.4)	9.21 (9.09–9.33)	288 (0.3)	0.13 (0.12–0.15)	<0.0001
SBP	9564 (8.8)	4.38 (4.30–4.47)	650 (0.6)	0.30 (0.27–0.32)	<0.0001
HCC	8996 (8.2)	4.12 (4.04–4.21)	270 (0.2)	0.12 (0.11–0.14)	<0.0001
Jaundice	2916 (2.7)	1.34 (1.29–1.38)	151 (0.1)	0.07 (0.06–0.08)	<0.0001
HRS	2441 (2.2)	1.12 (1.07–1.16)	54 (0.05)	0.02 (0.02–0.03)	<0.0001

Abbreviations: CI—confidence interval; SBP—spontaneous bacterial peritonitis; HCC—hepatocellular carcinoma; HRS—hepatorenal syndrome.

**Table 3:** Crude mortality rates per 100 person-years for non-alcoholic liver cirrhosis patients by etiology.

Cause of Death	A. HBV Only		B. HCV Only		C. HBV + HCV		D. NAFLD	
	*n* = 14,838		*n* = 11,861		*n* = 1425		*n* = 4148	
	(41,305.20 Person-Years)		(36,388.40 Person-Years)		(4035.95 Person-Years)		(15,281.62 Person-Years)	
	Deaths (*n*,%)	Rate (95% CI)	Deaths (*n*,%)	Rate (95% CI)	Deaths (*n*,%)	Rate (95% CI)	Deaths (*n*,%)	Rate (95% CI)
All-cause	6480 (43.7)	21.8 (21.4–22.3)	4882 (41.2)	20.6 (20.1–21.1)	596 (41.8)	20.9 (19.4–22.4)	1488 (35.9)	17.9 (17.1–18.8)
Variceal hemorrhage	3324 (22.4)	11.2 (10.8–11.6)	2590 (21.8)	10.9 (10.5–11.3)	299 (21.0)	10.5 (9.37–11.6)	823 (19.8)	9.92 (9.28–10.6)
Ascites	3049 (20.5)	10.3 (9.93–10.6)	2235 (18.8)	9.42 (9.05–9.79)	254 (17.8)	8.91 (7.87–9.96)	710 (17.1)	8.56 (7.96–9.16)
Encephalopathy	2630 (17.7)	8.86 (8.54–9.19)	2176 (18.3)	9.17 (8.81–9.54)	251 (17.6)	8.81 (7.77–9.85)	633 (15.3)	7.63 (7.06–8.20)
SBP	1336 (9.0)	4.50 (4.27–4.74)	1019 (8.6)	4.30 (4.04–4.55)	126 (8.8)	4.42 (3.67–5.18)	310 (7.5)	3.74 (3.33–4.14)
HCC	1988 (13.4)	6.70 (6.41–6.98)	1487 (12.5)	6.27 (5.96–6.58)	177 (12.4)	6.21 (5.32–7.10)	215 (5.2)	2.59 (2.25–2.93)
Jaundice	446 (3.0)	1.50 (1.36–1.64)	230 (1.9)	0.97 (0.84–1.09)	36 (2.5)	1.26 (0.85–1.67)	125 (3.0)	1.51 (1.24–1.77)
HRS	394 (2.7)	1.33 (1.20–1.46)	243 (2.0)	1.02 (0.90–1.15)	21 (1.5)	0.74 (0.42–1.05)	81 (2.0)	0.98 (0.76–1.19)

Abbreviations: HBV—hepatitis B virus; HCV—hepatitis C virus; NAFLD—non-alcoholic fatty liver disease; CI—confidence interval; SBP—spontaneous bacterial peritonitis; HCC—hepatocellular carcinoma; HRS—hepatorenal syndrome.

**Table 4:** Adjusted hazard ratios for all-cause mortality and mortality from different complications of non-alcoholic liver cirrhosis (vs. without non-alcoholic liver cirrhosis).

Variable	Event			
	Non-Alcoholic Liver Cirrhosis vs. Control			
	*n*	Adjusted HR *	95% CI	*p*-Value
(1) All-cause				
Etiology				
HBV only	7629	8.38	(7.87–8.93)	<0.0001
HCV only	6378	4.34	(4.09–4.60)	<0.0001
HBV + HCV	726	6.69	(5.53–8.10)	<0.0001
NAFLD	1997	3.80	(3.43–4.20)	<0.0001
Sex				
Female	18,081	4.70	(4.54–4.86)	<0.0001
Male	41,900	5.34	(5.22–5.47)	<0.0001
Age, years				
<40	3252	13.3	(11.9–15.0)	<0.0001
40–49	9133	9.66	(9.10–10.3)	<0.0001
50–59	12,562	7.33	(7.00–7.68)	<0.0001
60–69	15,918	4.72	(4.55–4.89)	<0.0001
≥70	19,116	3.24	(3.14–3.34)	<0.0001
(2) Variceal hemorrhage				
Etiology				
HBV only	3642	16.1	(14.3–18.0)	<0.0001
HCV only	2980	9.00	(8.09–10.0)	<0.0001
HBV + HCV	335	13.0	(9.15–18.3)	<0.0001
NAFLD	947	8.72	(7.22–10.5)	<0.0001
Sex				
Female	8315	11.7	(10.9–12.5)	<0.0001
Male	20,837	12.6	(12.1–13.2)	<0.0001
Age, years				
<40	1986	34.7	(27.9–43.2)	<0.0001
40–49	5460	28.9	(25.7–32.6)	<0.0001
50–59	6670	19.4	(17.7–21.3)	<0.0001
60–69	7436	11.4	(10.6–12.2)	<0.0001
≥70	7600	6.17	(5.83–6.53)	<0.0001
(3) Ascites				
Etiology				
HBV only	3112	72.0	(56.1–92.4)	<0.0001
HCV only	2310	39.9	(31.7–50.3)	<0.0001
HBV + HCV	259	74.5	(30.7–180.6)	<0.0001
NAFLD	721	83.3	(45.9–151.0)	<0.0001
Sex				
Female	7094	111.9	(91.1–137.5)	<0.0001
Male	16,057	87.0	(77.3–97.8)	<0.0001
Age, years				
<40	1643	94.5	(64.2–139.3)	<0.0001
40–49	4371	108.3	(84.2–139.1)	<0.0001
50–59	5535	112.6	(89.6–141.4)	<0.0001
60–69	5968	84.8	(70.1–102.6)	<0.0001
≥70	5634	79.5	(65.4–96.7)	<0.0001
(4) Encephalopathy				
Etiology				
HBV only	2698	58.0	(45.6–73.8)	<0.0001
HCV only	2231	53.0	(40.5–69.2)	<0.0001
HBV + HCV	256	75.0	(30.9–181.9)	<0.0001
NAFLD	643	83.0	(44.5–155.1)	<0.0001
Sex				
Female	6604	164.5	(127.3–212.6)	<0.0001
Male	13,791	92.0	(80.7–104.8)	<0.0001
Age, years				
<40	1493	112.5	(72.4–175.0)	<0.0001
40–49	4001	91.2	(71.7–116.1)	<0.0001
50–59	4853	125.7	(97.3–162.6)	<0.0001
60–69	5253	114.7	(90.8–145.0)	<0.0001
≥70	4795	99.0	(78.3–125.2)	<0.0001
(5) Spontaneous bacterial peritonitis				
Etiology				
HBV only	1440	19.9	(16.3–24.3)	<0.0001
HCV only	1113	14.8	(12.0–18.3)	<0.0001
HBV + HCV	132	32.4	(14.2–73.5)	<0.0001
NAFLD	336	15.9	(10.6–23.7)	<0.0001
Sex				
Female	3161	22.6	(19.6–26.1)	<0.0001
Male	7053	23.4	(21.2–25.7)	<0.0001
Age, years				
<40	797	55.4	(36.3–84.7)	<0.0001
40–49	2117	38.3	(30.9–47.7)	<0.0001
50–59	2555	27.6	(23.2–32.7)	<0.0001
60–69	2612	23.1	(19.7–27.0)	<0.0001
≥70	2133	12.3	(10.7–14.1)	<0.0001
(6) Hepatocellular carcinoma				
Etiology				
HBV only	2047	49.8	(38.4–64.6)	<0.0001
HCV only	1574	23.7	(19.1–29.5)	<0.0001
HBV + HCV	182	54.3	(22.3–132.4)	<0.0001
NAFLD	222	40.1	(18.9–85.2)	<0.0001
Sex				
Female	2618	75.1	(57.0–98.8)	<0.0001
Male	6648	46.2	(40.4–52.9)	<0.0001
Age, years				
<40	435	57.5	(31.6–104.7)	<0.0001
40–49	1404	66.5	(46.8–94.5)	<0.0001
50–59	2358	67.3	(51.4–88.2)	<0.0001
60–69	2721	50.3	(40.5–62.4)	<0.0001
≥70	2348	38.3	(30.9–47.4)	<0.0001
(7) Jaundice				
Etiology				
HBV only	480	19.9	(14.0–28.2)	<0.0001
HCV only	253	13.8	(8.97–21.2)	<0.0001
HBV + HCV	31	50.4	(6.90–367.7)	0.0001
NAFLD	130	32.9	(13.5–80.5)	<0.0001
Sex				
Female	741	29.4	(21.0–41.3)	<0.0001
Male	2326	30.0	(24.9–36.2)	<0.0001
Age, years				
<40	390	59.3	(31.6–111.1)	<0.0001
40–49	807	62.0	(39.8–96.7)	<0.0001
50–59	780	33.2	(23.7–46.7)	<0.0001
60–69	562	24.0	(17.0–33.8)	<0.0001
≥70	528	13.9	(10.4–18.6)	<0.0001
(8) Hepatorenal syndrome				
Etiology				
HBV only	407	42.7	(24.6–74.2)	<0.0001
HCV only	251	41.2	(20.4–83.3)	<0.0001
HBV + HCV	22	30.2	(4.05–225.3)	0.0009
NAFLD	84	34.3	(10.8–108.7)	<0.0001
Sex				
Female	683	109.1	(56.5–210.7)	<0.0001
Male	1812	59.3	(44.1–79.7)	<0.0001
Age, years				
<40	193	69.6	(25.8–187.4)	<0.0001
40–49	533	155.5	(64.4–375.1)	<0.0001
50–59	663	90.4	(49.9–164.1)	<0.0001
60–69	560	70.4	(39.7–124.8)	<0.0001
≥70	546	34.8	(22.7–53.4)	<0.0001

* Estimates were obtained using Cox’s proportional hazard Model with the adjustment of patient etiology, age, sex, CCI score, smoking status, and index date.

## Data Availability

Data is contained within the article.
